# Noninvasive Evaluation of Liver Fibrosis Reverse Using Artificial Neural Network Model for Chronic Hepatitis B Patients

**DOI:** 10.1155/2019/7239780

**Published:** 2019-07-21

**Authors:** Wei Wei, Xiaoning Wu, Jialing Zhou, Yameng Sun, Yuanyuan Kong, Xu Yang

**Affiliations:** ^1^Clinical Epidemiology and Evidence-Based Medical Center, National Clinical Research Center for Digestive Disease, Beijing Friendship Hospital, Capital Medical University, Beijing 100050, China; ^2^Liver Research Center, Beijing Friendship Hospital, Capital Medical University, Beijing Key Laboratory of Translational Medicine on Liver Cirrhosis, National Clinical Research Center for Digestive Disease, Beijing 100050, China; ^3^School of Computer Science and Technology, Beijing Institute of Technology, Beijing 100081, China

## Abstract

The diagnostic performance of an artificial neural network model for chronic HBV-induced liver fibrosis reverse is not well established. Our research aims to construct an ANN model for estimating noninvasive predictors of fibrosis reverse in chronic HBV patients after regular antiviral therapy. In our study, 141 consecutive patients requiring liver biopsy at baseline and 1.5 years were enrolled. Several serum biomarkers and liver stiffness were measured during antiviral therapy in both reverse and nonreverse groups. Statistically significant variables between two groups were selected to form an input layer of the ANN model. The ROC (receiver-operating characteristic) curve and AUC (area under the curve) were calculated for comparison of effectiveness of the ANN model and logistic regression model in predicting HBV-induced liver fibrosis reverse. The prevalence of fibrosis reverse of HBV patients was about 39% (55/141) after 78-week antiviral therapy. The Ishak scoring system was used to assess fibrosis reverse. Our study manifested that AST (aspartate aminotransferase; importance coefficient = 0.296), PLT (platelet count; IC = 0.159), WBC (white blood cell; IC = 0.142), CHE (cholinesterase; IC = 0.128), LSM (liver stiffness measurement; IC = 0.125), ALT (alanine aminotransferase; IC = 0.110), and gender (IC = 0.041) were the most crucial predictors of reverse. The AUC of the ANN model and logistic model was 0.809 ± 0.062 and 0.756 ± 0.059, respectively. In our study, we concluded that the ANN model with variables consisting of AST, PLT, WBC, CHE, LSM, ALT, and gender may be useful in diagnosing liver fibrosis reverse for chronic HBV-induced liver fibrosis patients.

## 1. Introduction

The progress to liver cirrhosis is a vital stage of chronic hepatitis B (CHB). Recent research demonstrated about 15–40% of CHB patients would progress to cirrhosis, liver failure, or hepatocellular carcinoma [1]. Persistent antiviral treatment for chronic HBV infection would suppress progress to cirrhosis and even implement reverse of fibrosis in the early stage [2].

Liver biopsy was considered the gold standard for distinguishing different stages in the diagnosis of liver cirrhosis, but the value was unstable because of invasiveness, sampling error, lack of standards, or intra- and interobserver agreement [3]. In recent years, noninvasive diagnosis models based on clinical and serological biomarkers for assessing liver cirrhosis have been calculated for CHB patients as the alternative marker to liver biopsy [4–6].

The correlations between serological biomarkers and the reverse of liver biopsy score are nonlinear and complex. Several research studies have explored the artificial neural network (ANN) model to estimate the correlation between serological biomarkers and the reverse of liver cirrhosis [7, 8]. ANNs base on the machine learning mechanism to identify the complex relationship between input neural units and output neural units. A systematic review suggested that ANNs were known to handle complex relationships better than linear statistic algorithms [8].

The aim of our study was to estimate the effectiveness of the noninvasive ANN model in estimating reverse of liver cirrhosis based on clinical variables and serological biomarkers.

## 2. Materials and Methods

### 2.1. Data Sources and Patients

In this study, we used the database from National Science and Technology Major Project, which enrolled 21 hospitals and 298 patients dating from July 2013 to December 2015. The demographic data and key laboratory data of patients were collected.

The enrollment criteria for this prospective study were as follows: treatment-naive patients with chronic HBV-induced fibrosis S2/S3 (similar to F2/F3, Ishak 2/3/4), who consented to undergo liver biopsy before and after treatment; patients who are HBeAg positive, HBVDNA >2 × 10^4^ IU/ml; or patients who are HBeAg negative, HBVDNA >2 × 10^3^ IU/ml.

**Figure 1:**
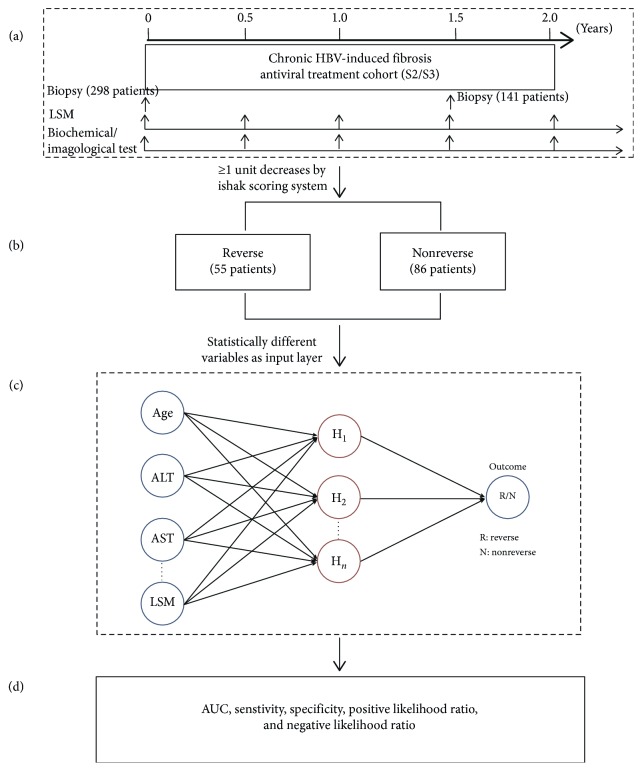
Flowchart of predicting liver fibrosis reverse factors and analytic method. (a) Summary of enrolled patients and relevant variables of reverse in our chronic HBV-induced fibrosis antiviral treatment cohort. (b) At the end of treatment, fifty-five patients reversed according to the Ishak scoring system. (c) Assessing predictors in the ANN model. Seven statistically different variables were pointed as the input layer, and the outcome was liver fibrosis reverse. (d) Evaluating diagnosis efficacy by AUC, sensitivity, specificity, positive likelihood ratio, and negative likelihood ratio.

One treatment group uses entecavir alone for 2 years, and the other group uses entecavir alone for the first 0.5 years and then entecavir plus pegylated interferon (peg-IFN) for 1 year and entecavir for another additional 0.5 years. At the end of treatment, all patients would undergo the second liver biopsy for assessing the liver fibrosis reversion. According to the Ishak scoring system, the decrease ≥1 unit was considered reverse after treatment (Figure 1).

All the patients agreed to follow-ups regularly, and the Ethics Committee of Beijing Friendship Hospital, Capital Medical University, approved the study protocol (BJFH 2014033).

### 2.2. Serological Biomarkers for Cirrhosis

Patients were assessed at baseline and at every six months for blood count, liver function test, HBVDNA, AFP (alpha-fetoprotein), PT (prothrombin time), thyroid function, liver ultrasonography, and Fibroscan. The second liver biopsy will be performed to evaluate the regression rate of liver fibrosis 1.5 years after initial therapy.

Reverse of liver cirrhosis was defined as the decrease of at least 1 point by the Ishak scoring system after 1.5 years compared with the baseline biopsy score.

### 2.3. Artificial Neural Networks

An artificial neural network consists of a set of processing units which simulate neurons and are interconnected via a set of weighted connections in a way which allows signals to travel through the network in parallel as well as serially [9].

The constructed ANN in this work consists of three layers. The input layer represents the observed results of serum biochemical and Fibroscan tests. The output layer is the indicator of reverse or nonreverse. The amplitude of the signal transmitted between neurons depends on the signal intensity emerging from the sending neuron and on the weight of their connection, the latter being denoted as the connection weight [10]. The three-layer network with a hidden layer was trained as follows:

(1) $$\begin{array}{c} \Delta_{p}\omega_{ji}=\theta \delta_{pj}o_{pi}, \end{array}$$

where *p* is the patient's number in the training dataset, each node could be abstracted to the corresponding state variable *x*_*j*_, and *ω*_*ji*_ is the weighted connections expressing the importance between node *i* and node *j*. The output node was calculated to indicate whether the weighted sum is less than or greater than a threshold *θ* value:

(2) $$\begin{array}{c} \delta_{pj}=\left(t_{pj}-o_{pj}\right)f_{j}\left({\text{Net}}_{pj}\right), \end{array}$$

in which each node included a transform function *f*_*j*_(*X*), *t*_*pj*_ is the expected output, and *o*_*pj*_ is the observed output. The output layer was expressed in formula (2) estimated by the transform function:

(3) $$\begin{array}{c} \delta_{pj}=f_{j}\left({\text{Net}}_{pj}\right)\sum_{k}\delta_{pk}\omega_{kj}. \end{array}$$

The hidden layer derived from input nodes was calculated in formula (3). Our network's output was limited to the binary set {0, 1} according to liver fibrosis reverse after 78-week antiviral therapy [11–13].

To provide a reasonable predictive model and comply with the standard way, our dataset was randomly divided into two subsets: 80% of the entire dataset for the training set and 20% for the testing set. The independent variables (input layer) were gender, PLT, WBC, ALT, AST, CHE, and LSM, which were statistically significant between the reverse and nonreverse groups. The dependent variable (output layer) was reverse or nonreverse after 1.5 years.

### 2.4. Statistical Analysis

Quantitative data were described as median, lower quartile, and upper quartile. And categorical data were described as frequency and percentage. Differences between the groups were compared using Student's *t*-test (normally distributed variables) or the Mann–Whitney test (nonnormally distributed variables) for continuous variables. The chi-square test or Fisher's exact test was used for categorical variables.

A three-layer feedback ANN model with the propagation algorithm was constructed according to the results of univariate and sensitivity analyses. Data were randomly divided into the training group (80%) and testing group (20%) in our exploratory ANN prediction model. Sigmoid transfer functions were performed in the hidden and output layers. Gradient descent was used for assessing connection weights. The overfit penalty was pointed as 0.001, and the convergence criterion was 0.00001 [14]. The outcome variable transformed to the range from 0 to 1 by the normalization algorithm. Liver fibrosis reverse was predicted if the outcome was equal to or greater than 0.5. SPSS modeler 18.0 (SPSS Inc., Chicago, IL, USA) was used for the ANN model.

A stepwise logistic regression analysis was performed to construct a logit model for comparison with our ANN model. The probability for conditional stepwise entry predictors was 0.05 and that for the removal predictors was 0.10. The ROC curve was calculated to assess the sensitivity, specificity, positive and negative likelihood ratios, accuracy, and area under the ROC curve (AUROC). These analyses were performed using SAS 9.2 (SAS Institute Inc., Cary, NC, USA). *P* values below 0.05 were considered significant.

## 3. Results

### 3.1. Distribution of Reverse and Nonreverse in CHB Patients after 1.5 Years of Therapy

A total of 298 patients with chronic HBV-induced fibrosis S2/S3 were enrolled in our study, and 141 patients consented to undergo liver biopsy after 1.5 years of antiviral therapy. About 39% (55/141) of patients were diagnosed reverse based on the Ishak scoring system (Figure 1).

### 3.2. Univariate Test Results for CHB Patients before and after Treatment

Our study found that HBVDNA (transformed to log_10_, *t* = 31.067, *P* < 0.001), PLT (*Z* = 2.700, *P*=0.007), WBC (*t* = 4.651, *P* < 0.001), ALT (*Z* = 15.555, *P* < 0.001), AST (*Z* = 15.387, *P* < 0.001), ALB (albumin; *t* = 4.711, *P* < 0.001), CHE (*Z* = 4.952, *P* < 0.001), TBIL (total bilirubin; *t* = 3.639, *P* < 0.001), PT (*t* = 7.046, *P* < 0.001), INR (international normalized ratio; *t* = 10.084, *P* < 0.001), AFP (*Z* = 7.220, *P* < 0.001), and LSM (*t* = 7.976, *P* < 0.001) were statistically different between baseline and 78 weeks, which are manifested in Table 1.

### 3.3. Multivariate Analysis of Reverse and Nonreverse Groups after 78 Weeks

At the end of 1.5 years, 141 patients undertook the second liver biopsy for assessing the curative effect. Fifty-five patients were diagnosed reverse and 86 patients were diagnosed nonreverse according to the Ishak scoring system. Seven variables were statistically significant and considered relevant to liver fibrosis reverse by univariate analysis: age (*X*^2^ = 4.059, *P*=0.044), PLT (*Z* = 3.478, *P* < 0.001), WBC (*t* = 3.744, *P* < 0.001), ALT (*t* = 1.988, *P*=0.048), AST (*t* = 3.060, *P*=0.003), CHE (*Z* = 3.217, *P*=0.001), and LSM (*t* = 2.024, *P*=0.043) (Table 2). A logistic regression was constructed to predict liver fibrosis reverse as follows: logit(*P*_reverse_)= 0.107+2.425 *∗* gender+1.024 *∗* ALT+1.009 *∗* PLT+0.997 *∗* CHE+0.980 *∗* WBC+0.945 *∗* AST+0.945 *∗* LSM.

### 3.4. ANN Analysis

**Figure 2:**
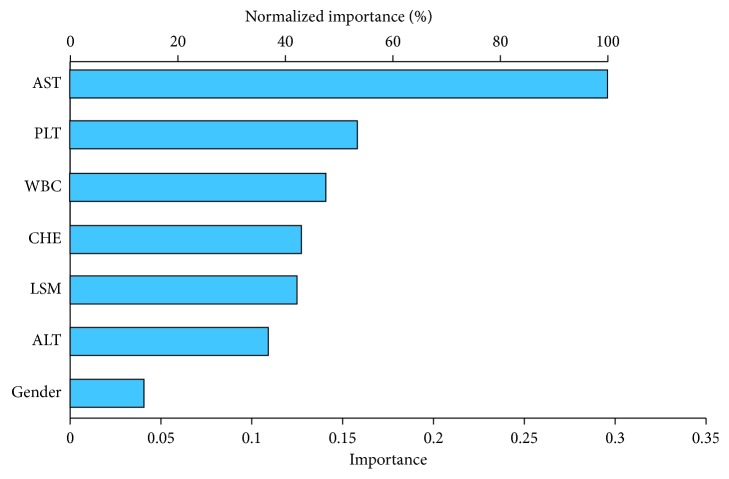
Predictive importance of the input variables. As demonstrated in the ANN model, AST, PLT, WBC, CHE, LSM, ALT, and gender were the most important predictors of liver cirrhosis reverse by sensitive analysis. AST was the most crucial node with the weight coefficient of 0.296 in our model.

**Figure 3:**
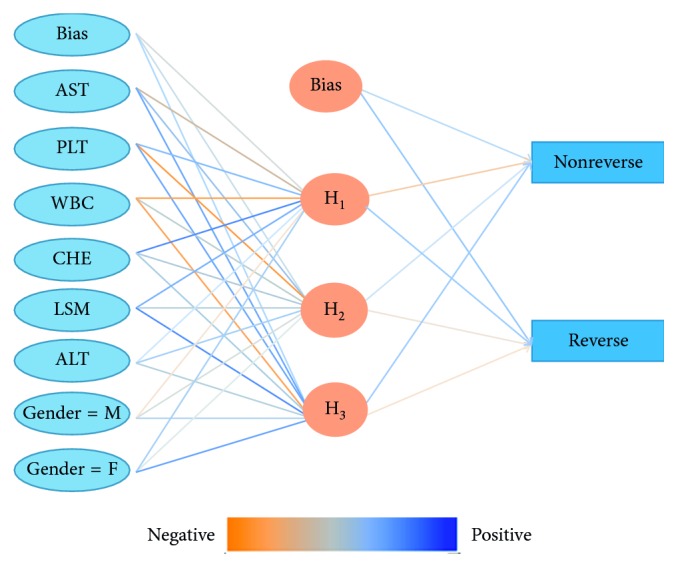
A three-layer neural network for the prediction of liver cirrhosis reverse. A feedforward backpropagation ANN model consisting of AST, PLT, WBC, CHE, LSM, ALT, and gender was constructed in 141 liver cirrhosis patients.

As manifested in Figure 2, AST, PLT, WBC, CHE, LSM, ALT, and gender were the most important predictors of liver biopsy reversion and assigned as the input layer in the ANN model. The importance coefficient of AST was 0.296 which contributed to the vital weight in our model. The three-layer feedback propagation neural network model included one input layer (containing 7 variables as inputs), one hidden layer (containing 4 neurons), and one output layer (Figure 3). The sensitivity, specificity, positive likelihood ratio, negative likelihood ratio, positive predictive value, and negative predictive value were 83.1%, 85.2%, 5.61, 0.19, 93.0%, and 74.5%, respectively, in the ANN model, which manifested better fitting function than logistic regression, as shown in Table 3.

## 4. Discussion

To our knowledge, this study is the first attempt to use the database of multicenter hospital-based HBV fibrosis patients with two liver biopsies to construct the ANN model for predicting fibrosis reverse after 1.5 years of antiviral therapy. Our results manifested AST, PLT, WBC, CHE, LSM, ALT, and gender were the noninvasive predictors of liver fibrosis reverse. According to ROC curve analysis and AUC analysis, the predictive performance of the ANN model was superior to the results of the logistic regression.

Compared to the classical statistic model based on the hypothesis that input variables and outcomes are linear regression relationships, the ANN is the machine learning algorithm based on the computational system, which could be constructed in a special structure to perform dynamic and continuous learning from knowledge as we input to the model [8]. ANNs have been used to predict the outcome events, including survival and exploring complex relationships between different surgical groups for complex medical decision-making [15]. The ANN used in our study is a standard backpropagation neural network in which the input variable received information from the data and transferred it to the output variable [16]. The stop criterion is recommended to build based on cross validation. It would monitor the error on an independent set of data and stop the training process when this error begins to increase.

In our research, noninvasive diagnosis models consisted of AST, PLT, WBC, CHE, LSM, ALT, and gender, which were statistically significant in univariate and multivariate analysis. Recently, APRI [17] and FIB-4 [18], consisting of ALT, AST, PLT, and age, were commonly used noninvasive serological predictors for fibrosis diagnosis. The WHO (World Health Organization) guidelines recommended that APRI and FIB-4 could be used for HBV-reduced fibrosis assessment in countries and regions with limited medical resources [19]. Furthermore, other noninvasive serum biomarkers were calculated for predicting cirrhosis in liver diseases. The Lok score performed well in predicting clinically significant portal hypertension using transient elastography (TE) [20]. The AST-to-ALT ratio was commonly used to elevate the alcoholic liver disease pattern in patients with hepatitis C who progressed to liver cirrhosis [21]. Yuanyuan Kong et al. found that baseline FIB-4 [18] and Ishak score [22] as well as baseline LSM, PLT, and ALB and their changes during the first 6 months could predict histological reversal in CHB patients on antiviral therapy [23].

However, Marcellin pointed out in their study that APRI and FIB-4 scores were not suitable for CHB patients for assessing hepatic fibrosis according to the Ishak stage, especially in gauging improvements in liver fibrosis following therapy [24]. Noninvasive assessment with either serum biomarkers or TE can be used to monitor improvement in liver fibrosis during antiviral therapy. The correlation of fibrosis improvement predicted by noninvasive measurement with histology has yet to be determined (evidence grading is B2 according to the GRADE system) [25]. Therefore, our study was aimed to construct the most accurate noninvasive predictors of HBV-induced fibrosis reverse.

One of the major limitations of our study was the limited hospital-based sample size, especially with consent to the second liver biopsy, which was the invasive assessment with certain risks in fibrosis patients after 1.5 years of antiviral therapy. In order to improve the predictive performance of our model, a total of 150 CHB patients with baseline biopsy could be collected as the validation dataset in ANN analysis.

In conclusion, the ANN model with serum biomarkers and Fibroscan test consisting of AST, PLT, WBC, CHE, LSM, ALT, and gender could be useful for assessing HBV-induced fibrosis reverse after the antiviral therapy.

## Figures and Tables

**Table 1:** Patient characteristics at baseline and 78 w.

	Baseline (*n* = 298)	78 w (*n* = 141)	*t*/*Z* value	*p*
Gender, *n* (%)				
** **Male	205 (68.8)	118 (73.8)	1.231	0.267
** **Female	93 (31.2)	42 (26.3)		
Age				
** **Mean ± SD	37.9 ± 10.8	36.2 ± 9.9	1.653	0.099
HBVDNA log_10_ (IU/ml)				
** **Mean ± SD	6.1 ± 1.9	1.4 ± 0.3	31.067	**<0.001**
ALT (U/L)				
** **Median (*P*_25_, *P*_75_)	82.7 (45.3, 160.8)	25.0 (17.0, 32.4)	15.555	**<0.001**
AST (U/L)				
** **Median (*P*_25_, *P*_75_)	52.0 (35.9, 93.0)	24.3 (19.0, 30.0)	15.387	**<0.001**
PLT (*E*9/L)				
** **Median (*P*_25_, *P*_75_)	168.0 (135.0, 202.0)	156.0 (117.0, 193.7)	2.700	**0.007**
WBC (*E*9/L)				
** **Mean ± SD	5.4 ± 1.5	4.7 ± 1.6	4.651	**<0.001**
ALB (g/L)				
** **Mean ± SD	43.0 ± 4.4	45.0 ± 4.2	4.711	**<0.001**
CHE (*μ*mol/L)				
** **Median (*P*_25_, *P*_75_)	3685.0 (7.2, 7243.0)	10.8 (8.2, 8412.3)	4.952	**<0.001**
TBIL (*μ*mol/L)				
** **Mean ± SD	17.4 ± 14.3	13.1 ± 5.9	3.639	**<0.001**
BUN (mmol/L)				
** **Mean ± SD	4.6 ± 1.2	4.7 ± 1.3	0.826	0.409
Cr (*μ*mol/L)				
** **Mean ± SD	68.4 ± 14.5	66.1 ± 17.8	1.492	0.136
PT (s)				
** **Mean ± SD	12.6 ± 1.4	11.7 ± 1.1	7.046	**<0.001**
LSM (kPa)				
** **Median (*P*_25_, *P*_75_)	8.7 (6.7, 13.7)	6.2 (5.2, 8.2)	7.976	**<0.001**

**Table 2:** Patient characteristics at 78 w between reverse and nonreverse groups.

	Reverse (*n* = 55)	Nonreverse (*n* = 86)	*t*/*Z* value	*p*
Gender, *n* (%)				
** **Male	35 (63.6)	68 (79.1)	4.059	**0.044**
** **Female	20 (36.4)	18 (20.9)		
Age				
** **Mean ± SD	36.4 ± 10.0	36.5 ± 9.8	0.058	0.953
HBVDNA log_10_ (IU/ml)				
** **Median (*P*_25_, *P*_75_)	1.3 (1.3, 1.3)	1.3 (1.3, 1.5)	1.197	0.231
ALT (U/L)				
** **Median (*P*_25_, *P*_75_)	22 (16, 32)	28 (19, 36)	1.988	**0.048**
AST (U/L)				
** **Median (*P*_25_, *P*_75_)	21 (18, 27)	27 (21, 32)	3.060	**0.003**
PLT (*E*9/L)				
** **Median (*P*_25_, *P*_75_)	182 (132, 211)	132 (104, 174)	3.478	**<0.001**
WBC (*E*9/L)				
** **Mean ± SD	5.2 ± 1.4	4.2 ± 1.6	3.744	**<0.001**
ALB (g/L)				
** **Mean ± SD	45.3 ± 3.8	44.8 ± 4.1	0.719	0.473
CHE (*μ*mol/L)				
** **Median (*P*_25_, *P*_75_)	7134.0 (9.5, 9371.0)	9.5 (7.4, 7244.0)	3.217	**0.001**
TBIL (*μ*mol/L)				
** **Mean ± SD	12.7 ± 5.7	13.6 ± 5.9	0.886	0.371
BUN (mmol/L)				
** **Mean ± SD	4.4 ± 1.0	4.7 ± 1.2	1.502	0.136
Cr (*μ*mol/L)				
** **Mean ± SD	65.1 ± 13.5	69.4 ± 12.6	1.882	0.062
PT (s)				
** **Mean ± SD	11.5 ± 0.9	11.5 ± 0.9	0.001	0.999
LSM (kPa)				
** **Median (*P*_25_, *P*_75_)	5.8 (4.8, 7.5)	6.6 (5.4, 8.9)	2.024	**0.043**

**Table 3:** Sensitivity, specificity, and AUC of the ANN model and logistic regression.

	Sensitivity (%)	Specificity (%)	AUC	LR+	LR−	PPV (%)	NPV (%)
ANN model	83.1	85.2	0.809 ± 0.062	5.61	0.19	93.0	74.5
Logistic regression	75.0	78.6	0.756 ± 0.059	3.50	0.32	87.2	61.1

LR+, positive likelihood ratio; LR−, negative likelihood ratio; PPV, positive predictive value; NPV, negative predictive value.

## Data Availability

We have registered our study in ClinicalTrials.gov, and all the study data should be submitted to the National Science and Technology after the last patient's follow-up in 2021. Then, we may share the data through ClinicalTrials.gov.

## Abbreviations

AFP: Alpha-fetoprotein
ALB: Albumin
ALT: Alanine aminotransferase
ANN: Artificial neural network
APRI: AST-platelet ratio index
AST: Aspartate aminotransferase
AUC: Area under the curve
CHB: Chronic hepatitis B
CHE: Cholinesterase
FIB-4: Fibrosis index based on four factors
HBV: Hepatitis B virus
INR: International normalized ratio
LSM: Liver stiffness measurement
PLT: Platelet count
PT: Prothrombin time
ROC: Receiver-operating characteristic
TBIL: Total bilirubin
TE: Transient elastography
WBC: White blood cell
WHO: World Health Organization.
